# Review: Pro-inflammatory cytokines and hypothalamic inflammation: implications for insufficient feed intake of transition dairy cows

**DOI:** 10.1017/S1751731119003124

**Published:** 2020-03

**Authors:** B. Kuhla

**Affiliations:** Institute of Nutritional Physiology “Kellner”, Institute for Farm Animal Biology (FBN), Wilhelm-Stahl-Allee 2, 18196 Dummerstorf, Germany

**Keywords:** late pregnancy, early lactation, signalling, brain, intake regulation

## Abstract

Improvements in feed intake of dairy cows entering the early lactation period potentially decrease the risk of metabolic disorders, but before developing approaches targeting the intake level, mechanisms controlling and dysregulating energy balance and feed intake need to be understood. This review focuses on different inflammatory pathways interfering with the neuroendocrine system regulating feed intake of periparturient dairy cows. Subacute inflammation in various peripheral organs often occurs shortly before or after calving and is associated with increased pro-inflammatory cytokine levels. These cytokines are released into the circulation and sensed by neurons located in the hypothalamus, the key brain region regulating energy balance, to signal reduction in feed intake. Besides these peripheral humoral signals, glia cells in the brain may produce pro-inflammatory cytokines independent of peripheral inflammation. Preliminary results show intensive microglia activation in early lactation, suggesting their involvement in hypothalamic inflammation and the control of feed intake of dairy cows. On the other hand, pro-inflammatory cytokine-induced activation of the vagus nerve transmits signalling to the brain, but this pathway seems not exclusively necessary to signal feed intake reduction. Yet, less studied in dairy cows so far, the endocannabinoid system links inflammation and the hypothalamic control of feed intake. Distinct endocannabinoids exert anti-inflammatory action but also stimulate the posttranslational cleavage of neuronal proopiomelanocortin towards β-endorphin, an orexigen promoting feed intake. Plasma endocannabinoid concentrations and hypothalamic β-endorphin levels increase from late pregnancy to early lactation, but less is known about the regulation of the hypothalamic endocannabinoid system during the periparturient period of dairy cows. Dietary fatty acids may modulate the formation of endocannabinoids, which opens new avenues to improve metabolic health and immune status of dairy cows.

## Implications

Insufficient increase in feed intake in the early lactation period of dairy cows results in increased body fat mobilisation and compromises animal health. Tissue remodelling processes such as growth of the mammary gland and rumen-intestinal tract or uterus involution and placenta retention are accompanied by tissue damages, which may facilitate the penetration and entrance of microbial endotoxins into the organs, thereby eliciting inflammatory responses. Both inflammatory conditions and excessive mobilisation of body reserves reduce feed intake in early lactation. Understanding the physiological and biochemical pathways of how inflammatory and metabolic signals regulate feed intake is crucial for developing feeding strategies minimising inflammation while increasing feed intake and health.

## Introduction

The period from late pregnancy over the course of calving is accompanied by a significant reduction in feed intake, causing the entrance into mild negative energy balance of dairy cows. Due to the enormous milk secretion in early lactation and the insufficient increase in feed intake, the negative energy balance becomes even more aggravated. Energy balance involving energy intake and energy expenditure is tightly regulated in a bidirectional communication between peripheral organs and the brain. The hypothalamus is one of the key regions of the brain regulating energy balance as it receives and integrates input signals from the periphery. Hypothalamic neurons may sense humoral substances, including nutrient-related metabolites, hormones and cytokines, but also integrate neural signals from other brain regions, the tongue or orinasal origin to adjust feed intake and energy expenditure. Excellent studies on the involvement of hypothalamic neuropeptides regulating feed intake in ruminants were reviewed recently (Sartin *et al.*, 2010; Clarke, 2014). However, inflammatory processes, as frequently occurring in various peripheral organs during the transition period of dairy cows (Bradford *et al.*, 2015), can interfere with the control of feed intake and energy balance. Not surprisingly, hypothalamic neurons that are able to sense humoral metabolites and endocrine signals may also detect pro-inflammatory cytokines, the latter considered as one of the most severe factors deregulating energy balance by shifting the homeostatic ‘set point’ and reducing feed intake. In this context, it is particularly important to understand how inflammatory processes in the periphery of transition cows signal their anorexic action to the hypothalamus. In addition, recent studies performed in rodents provided evidence that, apart from peripheral pro-inflammatory cytokines, hypothalamic glia cells participate in the regulation of peripheral metabolic functions (Reis *et al.*, 2015). Microglia may produce pro-inflammatory cytokines, thereby linking hypothalamic inflammation and disturbances in feed intake without requiring inflammatory signals from the periphery. There is lack of information about the regulatory role of glia during the periparturient period of dairy cows, but preliminary results suggest its participation in retarding the increase of feed intake during early lactation (data shown in the chapter ‘Inflammatory signalling to the brain via the humoral pathway’).

A further interface between the control of feed intake and inflammation is the endocannabinoid system of the hypothalamus. Numerous studies completed in rodents provide evidence on the hyperphagic and anti-inflammatory action of distinct endocannabinoids. Therein, the classical view of anorexic proopiomelanocortin (**POMC**) producing neurons was challenged, and the modulatory role of endocannabinoids favouring the production of the orexigenic β-endorphin from POMC was described (Koch *et al.*, 2015). Thus, the endocannabinoid system might have strong implications in the adaptation to early lactation of dairy cows by reducing lipolysis, increasing feed intake and ameliorating severe inflammation, but there is paucity of information on their contribution to key biological processes in dairy sciences.

Rather than attempting to summarise the current knowledge on metabolic and inflammatory changes during the transition period, the objective of the present review is to focus on the production of pro-inflammatory cytokines and their impact on neuroendocrine signalling involved in the regulation of feed intake a few weeks before and after calving. Other known involvements of reactive oxygen species (**ROS**), prostaglandins, chemokines or histamine in the interaction between inflammation and feed intake control will not be considered herein. The review outlines the sources of pro-inflammatory cytokines in late pregnancy and early lactation, summarises the action of peripheral pro-inflammatory cytokines on feed intake, taking also into account the interaction between body condition and inflammation. Considering this framework, inflammatory signalling to the brain via the vagus nerve and the humoral pathway is discussed with particular reference to the modulation of hypothalamic orexigenes and anorexigenes by pro-inflammatory cytokines and lipopolysaccharide (**LPS**). Finally, feeding strategies for reducing inflammation and increasing feed intake of cows are highlighted.

## Pro-inflammatory cytokine sources in late pregnancy and early lactation

The transition period of dairy cows is defined as ranging between 3 weeks before until 3 weeks after parturition and is characterised by major changes in metabolism, the endocrine and immune systems. Particular uterine and mammary gland tissue remodelling attract cells of the innate immune system, such as neutrophils, macrophages and dendritic cells (van Engelen *et al.*, 2009; Bentley *et al.*, 2015). Tissue remodelling and accompanied tissue damage facilitate the penetration and entrance of microorganisms in these organs. In response to tissue damage and microbial infection, immune cells invade into the uterine and the mammary gland during involution or parturition and produce high levels of pro-inflammatory cytokines such as interleukine-1β (**IL-1β**), IL-6 and tumour necrosis factor-α (**TNF-α**) (van Engelen *et al.*, 2009; Bentley *et al.*, 2015). These cytokines act locally in a paracrine fashion but are also released into circulation, thereby operating in an endocrine manner. Indeed, plasma concentrations of IL-1β, IL-6 and TNF-α have been shown to be 1.5- to five-fold higher prepartum compared to the early lactation period (Ishikawa *et al.*, 2004; Silva *et al.*, 2015; Trevisi *et al.*, 2015). However, plasma TNF-α and IL-6 are considered early markers of inflammation which decrease below the limit of assay detection in later stages of inflammation, thus explaining the often observed absence of increased pro-inflammatory cytokine concentrations in early lactation. Nevertheless, markers of later inflammatory stages, primarily positive acute phase proteins, accumulate in early lactation such as plasma C-reactive protein, haptoglobin or fibrinogen (Debski *et al.*, 2016), but summarising those is beyond the scope of the present review.

**Figure 1:**
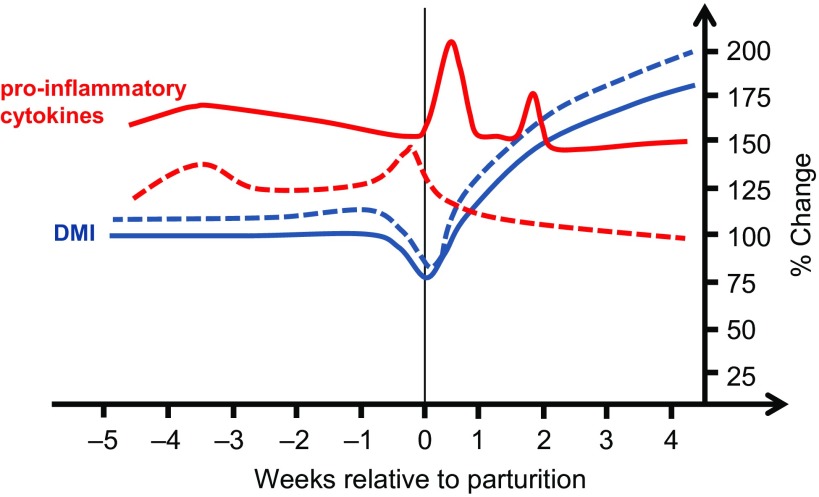
Schematic exemplary representing DM intake (DMI) in blue and systemic pro-inflammatory cytokine changes in red of cows with low (dashed line) or high (solid line) risk of inflammation during the periparturient period.

The level of serum pro-inflammatory cytokine concentrations varies enormously among studies analysing the periparturient period. One reason is the quality of commercial enzyme-linked immunosorbent assays, which may vary dramatically between manufactures, and many of them are poor quality kits with little or no validation. The lowest prepartum IL-6 serum concentrations for Holstein-Frisian dairy cows was measured by Trevisi *et al.* (2012), amounting to 120 to 350 pg/ml, whereas Ishikawa *et al.* (2004) reported concentrations between 1.5 and 3.5 ng/ml. Also, TNF-α and IL-1β serum concentrations determined on day 28 before calving varied quite substantially between cows and studies, ranging from 30 to 900 to 60 to 320 pg/ml, respectively (Trevisi et al., 2012, 2015). However, a general pro-inflammatory cytokine profile is illustrated in Figure 1. It is interesting to note that IL-6 serum concentrations did not increase in early and mid-pregnant sheep after LPS challenge, while they were highly responsive when the challenge was applied in late pregnancy and early lactation (Kabaroff *et al.*, 2006). The authors concluded an enhanced sensitivity of the hypothalamus–pituitary–adrenal axis producing cortisol during the transition period (Kabaroff *et al.*, 2006).

Bacterial infections of the mammary gland are most frequently detected during early lactation, but often results from contamination acquired during the preceding dry-off period in late pregnancy. Gram-positive and -negative bacteria may colonise the mammary gland, causing mastitis, but only gram-negative bacteria expose LPS in the outer cell wall. Several studies have shown that intramammary LPS may translocate into systemic circulation reaching five- to 18-fold higher plasma concentrations relative to non-infected cows (Hakogi *et al.*, 1989; Dosogne *et al.*, 2002). Moreover, LPS administration into the mammary gland increased plasma TNF-α concentrations by a factor of 1.9 and elevated hepatic mRNA expression of *Tnf* 2.5-fold, *Il1b* three-fold and *Il6* up to 15-fold (Vels *et al.*, 2009). Further sources of increased plasma LPS concentrations in early lactation are metritis and endometritis, both of which are characterised by a disrupted blood–uterine lumen barrier, increased plasma LPS and pro-inflammatory cytokine concentrations, the latter including IL-6 and TNF-α (Mateus *et al.*, 2003; Dervishi *et al.*, 2016). Also, retained placenta serves as a ground for bacterial growth in the uterus, but its retention seems to be caused by an impaired immune system and LPS tolerance developed before and around parturition (Eckel and Ametaj, 2016). Thus, cows with retained placenta show no significant increases in plasma pro-inflammatory cytokine concentrations postpartum (Trevisi *et al.*, 2015). Rather, concentrations <1 ng/ml of IL-6 were associated with retained placenta (Ishikawa *et al.*, 2004). Independent of placenta retention, essentially all cows have bacterial contaminations of the reproductive tract in the early postparturient period, which may contribute to single acute, chronic subacute, or accumulating inflammatory signals in the circulation (LeBlanc, 2010). Temporary plasma IL-6 spikes, approximately 1.5- to two-fold above the basal postparturient level, have been reported to occur, but they could not be attributed to postpartum uterus pathologies (Ishikawa *et al.*, 2004).

Although the rumen is almost not permeable for LPS when feeding diets based on roughage feed, under conditions of high portions of concentrate in the ration as they are usually applied after calving, ruminal LPS concentration increases whereas the pH decreases. The resulting acute or subacute rumen acidosis (**SARA**) harms the rumen epithelium, allowing LPS to translocate from the lumen into circulation (Khafipour *et al.*, 2009). The resulting inflammation of the rumen wall, called ruminitis, is associated with a 2.5- to five-fold higher mRNA and 1.5- to two-fold higher protein abundances of *Tnf*, *Il1b* and *Il6* genes in the ruminal epithelium (Zhao *et al.*, 2018). Finally, the same cows experiencing SARA showed three-fold higher TNF-α and 1.3-fold greater IL-6 serum concentrations, whereas IL-1β did not respond compared to healthy counterparts (Zhao *et al.*, 2018). Whether the increase in cytokine concentrations in the circulation originates mainly from blood macrophages, macrophages invading into the rumen wall, macrophagic (Kuppfer) cells in the liver when activated after portal LPS absorption, or a combination thereof is not yet resolved. Interestingly, cows with low liver functionality index show continuously increasing IL-6 serum concentrations in the early postparturient period (Trevisi *et al.*, 2012), pointing to the interaction between the level of concentrate feeding and hepatic immune response postpartum. Readers interested in the role of bacterial endotoxins and molecular mechanisms of inflammation in transition cows are referred to two recent excellent reviews by Bradford *et al.* (2015) and Eckel and Ametaj (2016).

## Peripheral pro-inflammatory cytokines and feed intake

Feed and thus energy intake of dairy cows is relatively constant between day 35 and day 10 prepartum and declines within the last week before calving by up to 20% (Kuhla *et al.*, 2015; Trevisi *et al.*, 2015). After parturition, DMI of cows increases reaching values of 170% to 200% relative to the prepartum period within the first 4 weeks of lactation (Figure 1). Thus, the pattern of feed intake is roughly considered inversely related to the periparturient pro-inflammatory cytokine profile; however, experiments testing the effects of peripherally administered pro-inflammatory cytokines, not LPS, on feed intake changes of dairy cows are rare. A single intravenous (i.v.) administration of recombinant bovine TNF (**rbTNF**) at a dosage of 5 µg/kg body weight (**BW**) or a continuous adipose tissue infusion of 14 μg/kg rbTNF per kilogram BW for 7 days to late-lactating Holstein cows did not alter DMI (Kushibiki *et al.*, 2002; Martel *et al.*, 2014), whereas daily subcutaneous administration of 3 µg rbTNF per kilogram BW for the first 7 days of lactation reduced DMI of Holstein cows by 24% and increased plasma TNF-α concentrations by a factor of 3 (Yuan *et al.*, 2013). This means that longer-term sustained, rather than short-term peak, TNF-α concentrations in the circulation and the stage of lactation seem to be crucial for exerting their influence on feed intake reduction. Although a few studies examined the physiological alterations after IL-1β and IL-6 administration in small ruminants (Herman et al., 2012, 2016; Wee *et al.*, 2011), their effects on feed intake have not been studied in depth. Therefore, the current knowledge about the role of IL-1β and IL-6 in controlling feed intake of cattle is restricted to association studies only. Trevisi *et al.* (2015) reported that dairy cows with greater area under the curve (**AUC**) levels of IL-1β and IL-6 between day 35 before until day 28 after parturition had lower DMI, both antepartum and postpartum, compared to counterparts with a lower periparturient AUC for IL-1β and IL-6. Ning *et al.* (2018) showed that i.v. infusion of 0.01 µg LPS per kilogram BW for ~6 h per day increased plasma IL-1β concentrations and decreased feed intake of mid-lactating Holstein cows. These findings suggest that increased circulating pro-inflammatory cytokine concentrations could be involved in signalling the reduction of feed intake during the periparturient period of dairy cows.

## Interaction between body condition score, inflammation and feed intake

Fat mass accreted within the last weeks or even months before calving is an important factor determining the level of feed intake before and after calving (Kuhla *et al.*, 2016). Adipose tissue reserves are mobilised during the periparturient period, providing glycerol and non-esterified free fatty acids (**NEFA**) as energy sources to other organs. Besides its metabolic function, adipose tissue is an active endocrine organ which secretes a range of cytokines regulating metabolism, feed intake and inflammation. A high body condition score (**BCS**), reflecting great amounts of stored adipose tissue, is associated with lower levels of feed intake, both antepartum and postpartum. This relationship can be explained among others by higher concentrations of leptin and a greater lipolytic capacity resulting in higher NEFA concentrations, both of which signal satiety in high BCS cows (Kuhla *et al.*, 2016). However, low DMI is not always associated with greater leptin serum concentrations (e.g. Roche *et al.*, 2005), suggesting that NEFA and leptin concentrations alone cannot explain the level of feed intake. This assumption is supported by the finding that the administration of 0.5 to 1.5 µg LPS or 5 µg rbTNF-α per kilogram BW did not affect plasma leptin concentrations in Holstein cows (Soliman *et al.*, 2002; Waldron *et al.*, 2003). On the other hand, Lippolis *et al.* (2017) showed that LPS infusion increased NEFA and leptin serum concentrations in beef cattle, further highlighting that in lactating dairy cows mechanisms other than NEFA and leptin alone cause a decrease in feed intake.

Intense lipolysis of adipose tissue generates local inflammatory responses, thereby participating in the regulation of the innate immune system during the transition period. By studying the mRNA expression in subcutaneous adipose tissue biopsies taken at days −10, +6 and +27 from 60 cows differing in BCS at dry-off, Vailati-Riboni *et al.* (2017) have shown that low BCS cows (4.25 on a 10-point scale) had lower postpartum mRNA expression of *Il6* and *Tnf*, the LPS receptor Toll-like receptor 4 (*Tlr4*) and the bacterial DNA receptor *Tlr9* compared to cows with a BCS of 5. These results correspond to the findings by Trevisi *et al.* (2012), who described higher serum IL-6 concentrations in cows with greater body fat mobilisation as indicated by higher plasma NEFA concentrations during the periparturient period. Also, fat ewes had greater plasma TNF-α concentrations than thin ewes (Daniel *et al.*, 2003). On the other hand, it has been reported from a study involving 40 lactating Holstein cows that animals with a very low BCS (2 to 2.5 on a five-point scale) had higher plasma TNF-α, IL-1β and IL-6 concentrations during early lactation than those with a more common BCS of 3 to 4 (Kasimanickam *et al.*, 2013). Unfortunately, these authors did not provide data on feed intake but suggested that perhaps increased pro-inflammatory cytokine concentrations mediate body condition loss in early lactation (Kasimanickam *et al.*, 2013). Indeed, elevated circulating concentrations of TNF-α, IL-1β and IL-6 have been described in patients with cachexia (Grossberg *et al.*, 2010). Although it is not clear to what extent the adipose tissue’s inflammatory response contributes to systemic pro-inflammatory cytokine concentrations, current data suggest that cows, being too thin or too thick before calving, are particularly prone to experience inflammation and that this response may participate in the control of feed intake.

## Inflammatory signalling to the brain via the vagus nerve

There are two major routes how visceral infection and inflammation is signalled to the brain: via the blood stream (humoral pathway) or by activating vagal afferents (neural pathway). The vagus nerve innervates large areas of the rumen-intestinal tract and contains both motor and sensory fibres. Sensory vagal afferents of the abdominal branch project from the viscera to the nucleus of the solitary tract, which is located in the brain stem and intensively interconnected with the hypothalamus. Both the number of vagal ascending fibres as well as their neurophysiological firing rate triggered by anorexigenic gut hormones was shown to determine the degree of satiety (Dockray, 2014). However, most of the studies examining the signal transduction of pro-inflammatory cytokines via the vagus nerve were performed in monogastric species. A systemic administration of low doses of IL-1β initiates the intestinal production of cholecystokinin (**CCK**), which in turn is well known as anorexigenic gut peptide, reducing feed intake in several species (Dockray, 2014). In cats it was shown that TNF-α enhanced the CCK-induced excitatory effect on leptin-sensitive vagus afferents (Quinson *et al.*, 2013). However, although CCK1 and IL-1 receptors are expressed at sensory vagal neurons and their ligand binding increases vagal discharge rate, subdiaphragmatic vagal deafferentation before intraperitoneal IL-1β or LPS administration suppressed food intake alike as in sham deafferentated rats (Porter *et al.*, 1998). Furthermore, surgical subdiaphragmatic vagotomy in sheep did not prevent anorexia, which in turn is observed in intact lambs 5 to 10 days after abomasal nematode (*Ostertagia circumcincta larvae*) infection (Fox *et al.*, 2006). Although the latter study did not examine whether the nematode infection led to increased pro-inflammatory cytokine secretions, the finding contributes to the current understanding that vagal sensory afferents can be activated by peripheral pro-inflammatory cytokines or LPS, but this activation is not exclusively necessary for their feed intake-suppressive effects. Pro-inflammatory cytokine- or LPS-induced activation of vagal afferents rather triggers an anti-inflammatory reflex leading to reduced intestinal inflammation and disease activity (Griton and Konsman, 2018). Conclusively, the vagus nerve plays a major role in sensing and combating visceral inflammation, but the humoral pathway by which pro-inflammatory cytokines transmit their information to the brain is of central importance for satiety signalling.

## Inflammatory signalling to the brain via the humoral pathway

As peripherally administered LPS can normally not cross the blood–brain barrier (**BBB**), its anorexigenic action is mediated indirectly by pro-inflammatory cytokines produced either in the periphery or the brain. Due to its relatively high molecular weight (>17 kDa), pro-inflammatory cytokines cannot simply penetrate the functioning of BBB. However, when radioactively labelled ovine IL-1β or IL-6 were injected into the jugular vein of mice, the brain/serum ratios increased linearly with exposure time, demonstrating that these pro-inflammatory cytokines cross at least some parts of the BBB by a saturable transport process (Threlkeld *et al.*, 2010). The four sensory circumventricular organs of the brain, specifically the vascular organ of lamina terminalis, the subfornical organ, the median eminence and the area postrema of the brainstem, do not form a functional BBB but consist of highly permeable capillaries allowing the transport of cytokines into adjacent brain areas and the third brain ventricle filled with cerebrospinal fluid (**CSF**). Pro-inflammatory cytokines transmitted from the blood to the brain initiate the production of central pro-inflammatory cytokines, thereby amplifying their own synthesis in the brain. The autostimmulatory effect of IL-1β is the best studied so far, and its role during disease-associated anorexia and cachexia was intensively reviewed (Konsman and Dantzer, 2001; Grossberg *et al.*, 2010). Central IL-1β production is mainly achieved by astroglial and microglial, but also perivascular and meningeal macrophages and some neurons (Konsman and Dantzer, 2001; Grossberg *et al.*, 2010). The neuronal cell bodies producing cytokines are primarily located in the periventricular region of the hypothalamus. On the other hand, microglial but not neuronal cells express TLR4, thereby mediating the effect of LPS on microglial nuclear factor-κB (**Nf-κB**) signalling and cytokine production (Reis *et al.*, 2015). When 250 µg LPS was administered into the third brain ventricle of sheep, *Tlr4* and *Il1b* mRNA expression was found increased in the mediobasal hypothalamus, whereas pre-treatment with a CD14/TLR4 antagonist reduced LPS-induced increase in *Il1b* mRNA abundance (Haziak *et al.*, 2018). Besides LPS, palmitic and stearic acid, which are increasingly released from adipose tissue of dairy cows during early lactation, have been shown to activate the TLR4 pathway, including phosphorylation and nuclear translocation of the p65 subunit of NF-κB and the production of pro-inflammatory cytokines in microglia (Wang *et al.*, 2012).

**Figure 2:**
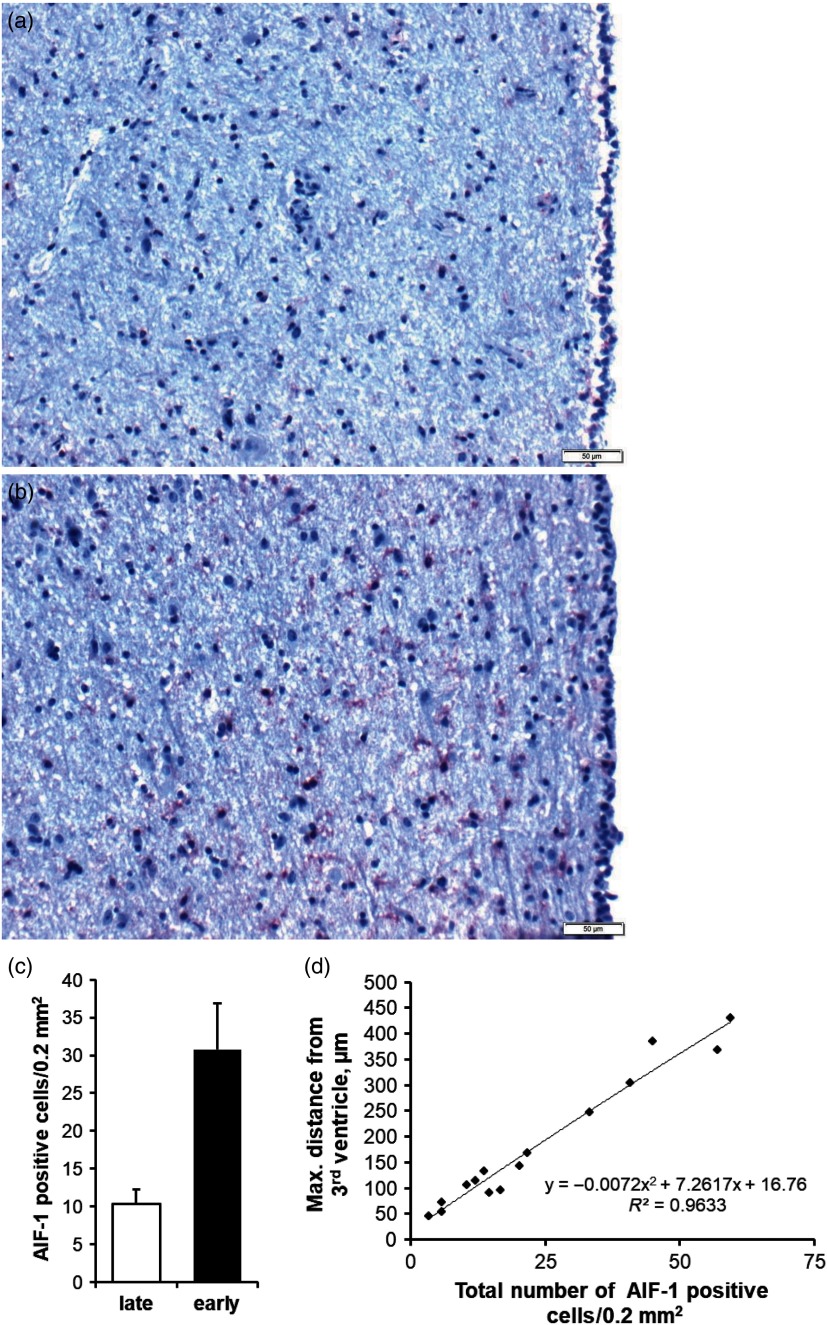
Microglia activation in the periventricular region of the hypothalamus of late- (a) and early-lactating (b) dairy cows. Activated microglia was immunostained for allograft inflammatory factor 1 (AIF-1) and visualised in red; cell nuclei were counterstained by haematoxylin (blue). Epithelial cells of the third ventricle are located at the right image margins. The scale bar indicates 50 µm. The number of activated microglia in the periventricular hypothalamic region in up to 500 µm radial distance from the third ventricle epithelium during early and late lactation is given as mean ± SE (c). Exponential relationship and coefficient of determination (*R*^2^) between the maximal distance of an AIF-1-positive cell from the third ventricle border and the total number of AIF-1-positive cells (d). Adapted from B. Kuhla (unpublished data).

The activation of microglia due to inflammatory signals is characterised, among others, by increased expression of the allograft inflammatory factor 1 (**AIF1**), which is also known as ionised calcium-binding adapter molecule 1 (**IBA1**). We analysed the abundance of AIF1 in the hypothalamus of five non-pregnant, late-lactating Holstein dairy cows (180 to 325 days in milk, second to fourth lactation) in positive energy balance and 10 early-lactating cows (11 to 66 days in milk, third and fourth lactation) in negative energy balance using a goat antibody from abcam (ab5076). Activated microglia was 2.8 times more abundant during early compared to late lactation (Figure 2a–c). In late lactation and positive energy balance, the few activated microglial cells in the periventricular region were predominantly found in short distance from the third ventricle border, whereas in early lactation and negative energy balance, the maximal distance was much greater. Thus, the longer the maximal distance of an AIF1-positive cell from the third ventricle epithelium, the higher the total number of periventricular AIF1 cells (Figure 2d). However, we found no direct relationship between the number of activated hypothalamic microglia and plasma TNF-α concentrations but instead a positive correlation between the number of AIF1 cells and plasma NEFA concentrations (unpublished data), suggesting that metabolic imbalances, perhaps in concert with ROS and other mediators, trigger hypothalamic inflammation in early lactation. Hypothalamic cytokine expression is also responsive to feed deprivation. A microanalysis of hypothalamic tissue extract revealed that mRNA expression of *Il1a* and the chemokine *Cxcl10* was significantly upregulated after 18 days of feed restriction relative to *ad libitum* feeding of anovulatory but not ovulatory Charolais-crossbreed heifers (Matthews *et al.*, 2017).

One upstream regulator of Nf-κB-regulated cytokine production is, besides others, the p38 mitogen-activated protein kinase (**p38 MAPK**). Indeed, the blockage of p38 MAPK by i.v. administration of the inhibitor SB203580 for 7 days abolished the 1.5- to two-fold increase in hypothalamic *Il1b, Il6* and *TNF* expression after i.v. LPS injections (400 ng/kg BW) to anoestrous ewes (Herman *et al.*, 2014). Even 1 h after i.v. administration of 1 µg LPS per kilogram BW, mRNA expression of leukemia inhibitory factor (***Lif***), an IL-6 class cytokine, was found two-fold higher in the arcuate nucleus (**ARC**) of the hypothalamus of sheep compared to vehicles (Daniel *et al.*, 2016). Moreover, intracerebroventricular (**i.c.v.**) administration of 2.5 µg LIF reduced cumulative feed intake of sheep 6 to 24 h post-injection relative to saline-treated controls (Daniel *et al.*, 2016). Also, i.v. administration of 40 µg LPS or i.c.v. administration of 10 µg IL-1β into the lateral brain ventricle of sheep increased *Il1b* and *cfos* mRNA expression in the paraventricular nucleus (**PVN**) of the hypothalamus as assessed by *in situ* hybridisation (Vellucci *et al.*, 1995; Vellucci and Parrott, 1996). Together, these findings suggest that peripheral inflammatory conditions, as frequently occurring during the transition period of dairy cows, result in increased production of hypothalamic pro-inflammatory cytokines at least during early lactation. Yet, it is unknown if induced hypothalamic inflammation in early lactation affects neuronal signalling controlling feed intake, or whether, due to central pro-inflammatory cytokine resistance, the reductive effect on feed intake is blunted. The latter assumption should be considered since McMahon *et al.* (1999) reported that i.c.v. infusion of a recombinant human IL-1 receptor antagonist (**IL-1ra**) did not prevent the reduction of feed intake of sheep subcutaneously infused with 20 µg LPS per kilogram BW. Furthermore, it must be noticed that molecules other than peripheral pro-inflammatory cytokines, that is, reactive oxygen species, histamine, toxins or nutrient deficiency, may also activate hypothalamic microglia, but the review of their actions is beyond the scope of the present paper.

## Hypothalamic orexigenes and anorexigenes and their modulation by pro-inflammatory cytokines and endotoxin

**Figure 3:**
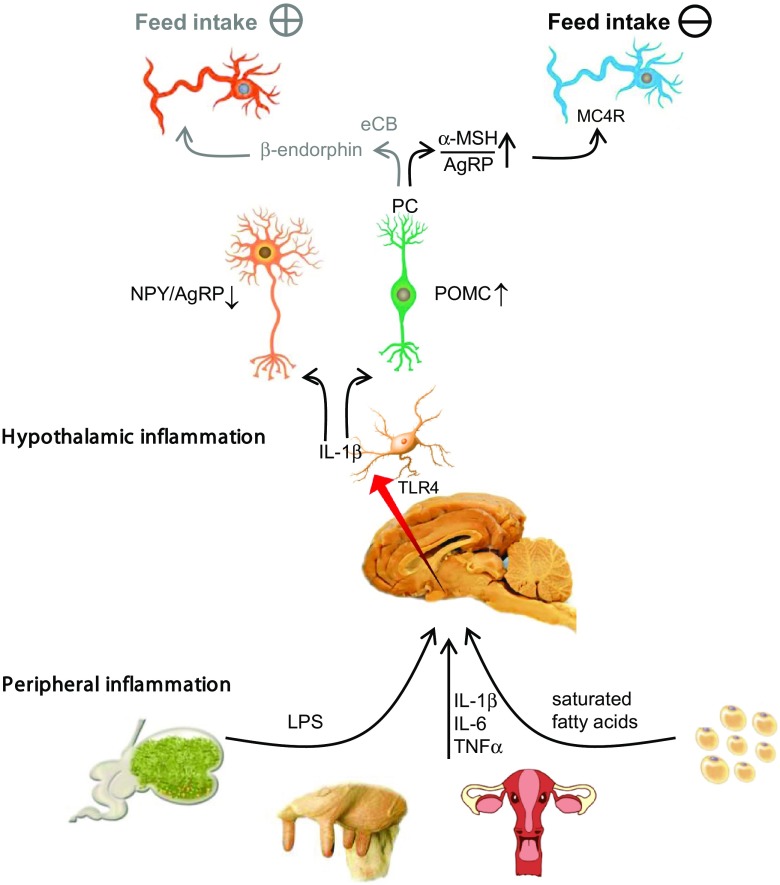
Black arrows illustrate the pathway on how peripheral pro-inflammatory cytokines, LPS or saturated fatty acids signal to induce hypothalamic inflammation, thereby diminishing the increase of feed intake during early lactation of dairy cows. Clinical or subclinical diseases such as ruminitis, mastitis or metritis, which often occur during the periparturient period, are characterised by elevated lipopolysaccharide (LPS) and pro-inflammatory cytokine concentrations in the circulation. Particularly during early lactation, the blood concentration of long-chain fatty acids is increased as well. Peripheral pro-inflammatory cytokines, such as interleukine-1β (IL-1β), IL-6 and tumour necrosis factor-α (TNF-α), but also saturated fatty acids, may cross permeable capillaries of the blood–brain barrier. LPS and saturated fatty acids can be detected by toll-like receptor 4 (TLR4), which is mainly expressed by astro- or microglial cells and signals to produce local pro-inflammatory cytokines. Thus, both peripheral and centrally produced pro-inflammatory cytokines can be detected by hypothalamic first-order neurons located in the arcuate nucleus. Locally increased pro-inflammatory cytokine concentrations in the hypothalamus activate proopiomelanocortin (POMC) neurons. Post-translational cleavage of POMC by PC increases α-melanocyte stimulating hormone (α-MSH) production, resulting in increased α-MSH–Agouti-related peptide (AgRP) ratio at second-order neurons expressing the melanocortin-4 receptor (MC4R), thereby causing a signal reduction in feed intake. Under absent or low inflammatory conditions (grey arrows), endocannabinoids (eCB) facilitate the production of β-endorphin from POMC, thereby increasing orexigenic signalling and feed intake during early lactation. NPY = neuropeptide Y.

The hypothalamus is one of the most important brain areas involved in sensing and integrating peripheral and central inputs to stimulate or repress feed intake and energy expenditure. In the ARC of the hypothalamus are at least two neuronal populations located which either convert input signals to promote (orexigenic) or inhibit (anorexigenic) feed intake. Because of their ability to detect peripheral hormones and metabolites, ARC neurons are often called first-order neurons. Orexigenic ARC neurons synthesise either neuropeptide Y (**NPY**) or Agouti-related peptide (**AgRP**), whereas anorexigenic ARC neurons produce cocaine-amphetamine-regulated transcript (**CART**), but more abundantly POMC. Post-translational enzymatic cleavage of the POMC protein by prohormone convertases (**PC**) results in numerous biologically active peptides of which predominantly α-melanocyte stimulating hormone (**α-MSH**) exerts anorexigenic function. However, POMC may also be cleaved, resulting in the formation of β-endorphin which elicits orexigenic effects. Further post-translational modifications, such as acetylation, modulate the binding intensity of these two peptides to their receptors, thereby altering their biological activity. While β-endorphin, as an endogenous opiate, is detected by µ-opioid receptors primarily abundant in outer-hypothalamic neurons, α-MSH binds to its melanocortin-4 receptor (**MC4R**), which is expressed by second-order neurons located in the lateral (**LH**) and ventromedial (**VMH**) hypothalamus. As a natural MC4R antagonist, AgRP competes for receptor binding with α-MSH, thereby attenuating the anorexigenic effect of α-MSH (Figure 3). These neuronal pathways are best described for rodent species, but were also confirmed in ruminants (Sartin *et al.*, 2010; Clarke, 2014). The expression of feed intake-controlling genes may differ in animals with divergent feed efficiency. Cardoso *et al.* (2015) reported that *POMC* mRNA expression in the ARC of heifers gaining >1 kg BW per day was greater than in counterparts gaining only 0.5 kg/day. Conversely, *NPY* mRNA expression was lower in these animals, although the authors did not present data on feed intake (Alves *et al.*, 2015). In the ARC of Angus cattle with a higher plane of DM intake (high residual feed intake (**RFI**)), *POMC* mRNA expression was also greater, while *NPY* and *MC4R* mRNA abundances were lower than in cattle with low RFI (Perkins *et al.*, 2014). Even though the abundances of hypothalamic pro-inflammatory cytokines and their receptors were not investigated in these studies, a recently performed gene network analysis showed that low-RFI cattle possess a heightened degree of inflammation, at least in the gut (Weber *et al.*, 2016).

Pro-inflammatory cytokine receptors such as IL-1RI and LIF-R are densely expressed in the ARC, and both POMC and AgRP neurons express IL-1 R (Grossberg *et al.*, 2010). Accordingly, i.c.v. administration of IL-1β into the lateral brain ventricle of rats induced transcriptional activity in POMC and also AgRP and NPY neurons (Grossberg *et al.*, 2010). Also, peripheral LPS administration to rats or sheep increased *Agrp* mRNA expression, and cFos activated AgRP cells above control levels (Grossberg *et al.*, 2010; Daniel *et al.*, 2016), but surprisingly, cFOS activated α-MSH-producing cells and POMC gene expression was reduced 6 h after sheep were injected with LPS (Sartin *et al.*, 2008). The authors hypothesised that a decrease in α-MSH and POMC and an increase in AgRP represents an adaptive response to an LPS-induced decrease in feed intake (Sartin *et al.*, 2008), and showed later that POMC expression did not differ 1 h after LPS treatment relative to controls (Daniel *et al.*, 2016). However, AgRP expression is increased already 1 h after LPS administration (Daniel *et al.*, 2016), which might be explained by another physiological function of the MC4R system, such as its anti-inflammatory action in the brain (Lasaga *et al.*, 2008). However, the anti-inflammatory action of α-MSH and MC4R has not been studied yet in transition dairy cows.

In contrast to the effect of pro-inflammatory cytokines on hypothalamic neuropeptides, a recent study by Reis *et al.* (2015) showed that LPS inhibited the firing rate of AgRP/NPY neurons, whereas it increased the activity of POMC neurons in brain explants from rodents and that these effects were abolished by blocking TLR4 receptors. Another study in mice has demonstrated that TLR2 activation by i.c.v. injection of a synthetic TLR2 agonist triggered hypothalamic inflammation by the activation of the microglial NF-κB signal pathway in the ARC, which resulted in an increased activity of POMC neurons, increased α-MSH fibres in the hypothalamic PVN, and anorexia (Jin *et al.*, 2016). In this publication, the authors further showed that administrations of NF-κB or MC4R antagonists reversed the anorexia induced by TLR2 activation. Also, the deactivation of microglial activity by i.c.v. injection of minocyclin hydrochloride increased feed intake of rats after 4 h (Reis *et al.*, 2015). These recent findings highlight the meaning of the microglia–POMC neuron axis in reducing feed intake during inflammatory states (Figure 3).

For dairy cows, fewer studies on hypothalamic neuropeptide signalling have been performed. Immunostaining for β-endorphin produced by POMC neurons located in ARC was greater for cows in late than in early lactation (Leshin *et al.*, 1992). Unfortunately, the latter study provided no data on the level of feed intake, but usually feed intake of dairy cows is greater in months 12 to 14 than between days 10 and 16 postpartum, suggesting a direct relationship between β-endorphin and feed intake in dairy cows. Also, other opiate peptides such as enkephalins and the POMC-processing enzyme PC2 were increased in CSF in early lactation relative to the prepartum period (Kuhla *et al.*, 2015). Data show that post-translational POMC processing and accompanied β-endorphin production is highly regulated during the periparturient period, possibly aligned to a signal increase in feed intake in early lactation. This idea is supported by the study of Obese *et al.* (2007) demonstrating that i.v. administration of the opiate syndyphalin-33 (0.05 to –0.1 µmol/kg BW), a µ-opioid receptor ligand, increased feed intake, whereas naloxone (1 mg/kg BW), an opioid antagonist, reduced feed intake of sheep. However, syndyphalin-33 did not prevent the reduction of feed intake induced by LPS administration (Obese *et al.*, 2007). Also, i.c.v. administration of dynorphin A prevented feed intake-reducing effect elicited by rumen distension or intraruminal propionic acid infusion (Della-Fera *et al.*, 1990).

**Figure 4:**
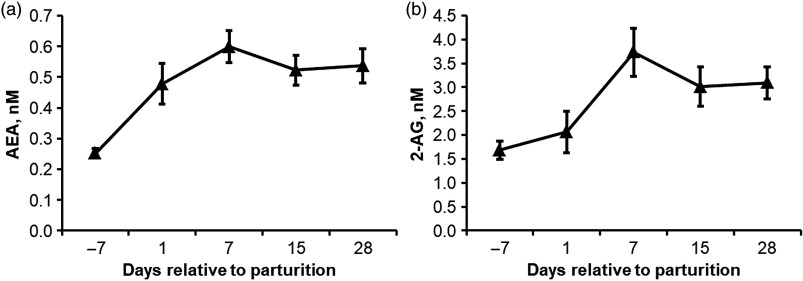
Plasma *N*-arachidonoylethanolamine (AEA) (a) and 2-arachidonoylglycerol (2AG) (b) concentrations in 16 periparturient dairy cows. The concentrations of both endocannabinoids was two- to 2.3-fold higher during early lactation (days 7 to 28) compared to the antepartum level (day 7) (*P* < 0.05). Data are shown as mean ± SE. Adapted from Kuhla *et al.* (2019).

Only recently it has been demonstrated that the production of β-endorphin from POMC is promoted by endocannabinoids (**eCB**), such as *N*-arachidonoylethanolamine (**AEA** or anandamide) and 2-arachidonoylglycerol (**2-AG**), thereby converting classically considered anorexic POMC neurons into drivers of feed intake (Koch *et al.*, 2015). We analysed the concentrations of AEA and 2-AG in the plasma of 16 Holstein dairy cows during the periparturient period by HPLC/MS and found that the concentrations of both eCB increased from day 7 before until day 7 after parturition 2.3-fold and sustained two-fold elevation thereafter at least by week 4 of lactation (Figure 4). The concentration of circulating and hypothalamic eCB may not be directly related, but if so, these results imply that the elevated eCB tone may account for increased β-endorphin production in POMC neurons during early lactation. Hence, future studies should examine hypothalamic eCB concentrations at different planes of feed intake and before and after parturition. Most interestingly, subcutaneous AEA administration attenuated LPS-induced hypophagia and decrease in hypothalamic cFOS expression in rats. Vice versa, the blockade of the endocannabinoid receptor 1 with the chemical compound AM251 increased LPS-mediated inflammatory effects in the hypothalamus (Surkin *et al.*, 2017), supporting the idea on eCBs being an opponent of inflammogenes (Hollis *et al.*, 2011).

The involvement of pro-inflammatory cytokines or TLR4 signalling in the regulation of neuropeptidergic circuits in cows has not been systematically studied yet. In heifers, a strong direct association between neuroinflammatory pathway activation and hypothalamic POMC was documented. The mRNA expressions of both *Tlr6* and *Pomc* were upregulated, whereas *Agrp* and *Npy* abundances were reduced in ARC of high-BW-gain heifers fed high-concentrate diet compared to low-BW-gain heifers fed a high-forage diet (Allen *et al.*, 2012). Unfortunately, expression data shown in the latter study were not related to feed or energy intake. In early-lactating dairy cows with a high extent of fat mobilisation, which, as described above, usually differ in their pro-inflammatory cytokine concentrations from less-mobilising counterparts, the percentage of cFOS-activated AgRP neurons was lower than in less-fat-mobilising cows (Börner *et al.*, 2013). These preliminary results suggest that, besides fatty acids or leptin, inflammatory conditions may also contribute to affect hypothalamic orexigenic and anorexigenic signalling in early lactation.

## Feeding strategies to reduce inflammation and increase feed intake

Various plant compounds have been shown to exert antioxidative and anti-inflammatory properties. These properties are attributed to increased contents of unsaturated, mainly n-3, fatty acids, distinct amino acids, polyphenolic flavonoids, carotenoids, vitamins or minerals. Increased intake of n-3 fatty acids from 2% fish or flax oil supplementation has been shown to reduce *ex vivo* LPS-induced TNFA or IL-4 gene expression in blood cells of Holstein calves, whereas this effect was not observed when pork fat was added to the milk replacer (Karcher *et al.*, 2014). Various studies testing the effect of different portions of unsaturated fatty acid supplementations to the diet of periparturient dairy cows have been performed, but not all reported an improvement in DMI, which could primarily be attributed to either too overdosing or insufficient rumen protection. Supplementing the diet with 4% to 7% whole flaxseed or whole linola on a DM basis between week 4 before and week 14 after calving increased DMI relative to feeding a diet isoenergetically formulated with calcium salts of palm oil (do Prado *et al.*, 2016). However, there was no effect of unsaturated fatty acid supplementation on the hepatic activities of superoxide dismutase and glutathione peroxidase, reflecting oxidative stress load, but unfortunately, cytokine expressions were not reported (do Prado *et al.*, 2016). Prepartum feeding of sunflower seeds rich in linoleic acid (C18:2) increased postpartum feed intake compared to canola seed (rich in oleic acid (C18:1)) supplementation, but did not affect uterine inflammation examined at day 25 postpartum (Salehi *et al.*, 2016). Moreover, reducing the n-6–n-3 polyunsaturated fatty acid ratio from 6 to 4 in the diet during the early lactation period (10 to 100 days in milk) increased DMI and reduced plasma IL-6 concentrations after intramammary gland LPS challenge on day 75 postpartum (Greco *et al.*, 2015). Interestingly, polyunsaturated fatty acids are detected by the G-protein-coupled receptors 40 (**GPR40**) and 120 (**GPR120**). While GPR40 is primarily expressed by POMC and NPY neurons, GPR120 was found at the cell surface of microglia (Dragano *et al.*, 2017). A combined activation of both receptors in the hypothalamus reduced the expression of markers characterising hypothalamic inflammation but also energy efficiency of obese mice (Dragano *et al.*, 2017). Thus, it seems that polyunsaturated fatty acids, at least to some degree, may restrict the formation of inflammation and improve DMI of early-lactating dairy cows.

Supplementation of 79 mg rumen-protected niacin per kilogram BW to the diet from 3 weeks before until 1 week after calving increased the abundances of liver mRNA coding for the acute inflammatory response, while this condition was not associated with an altered negative energy balance in dairy cows (Ringseis *et al.*, 2019). On the other hand, supplementing the ration with polyphenol-rich grape seed and grape marc meal extract (10 g/kg DM) from 3 weeks antepartum until 9 weeks postpartum reduced hepatic inflammatory processes and reduced the plasma concentrations of acute phase proteins haptoglobin and amyloid A of dairy cows, but again the amelioration of the inflammatory state was not accompanied by an improvement in feed intake or energy balance (Gessner *et al.*, 2017). However, whether the concentrations of pro-inflammatory cytokines were affected by niacin or grape meal supplementations was not reported in these studies.

Li *et al.* (2016) showed that rumen-protected methionine supplementation to a diet containing an energy level of NE_L_ = 1.54 Mcal/kg DM dampened the upregulation of hepatic *Tlr4* mRNA expression and increased DMI of early postparturient dairy cows, whereas this effect could not be observed when cows were fed a ration containing 1.24 Mcal/kg DM. The results imply that dietary supplementation of rumen-stable anti-inflammatory or antioxidative compounds is particularly effective on a high-energy-diet background which might explain why other studies failed to reduce pro-inflammatory cytokine expressions and improve feed intake of periparturient cows.

## Conclusions

The work reviewed suggests that insufficient feed intake during the transition period of dairy cows is a response to various inflammatory conditions prevailing in peripheral organs during this time. Peripheral pro-inflammatory cytokines, including TNF-α, IL-1β or IL-6, which are primarily released by macrophage-like cells into the circulation, are thought to be important mediators of hypophagia and hypothalamic inflammation, although the latter may arise independently of peripheral inflammogenes. The vagus nerve can transmit signalling triggered by peripheral pro-inflammatory cytokines or LPS from the periphery to the brain, but this communication line is not solely necessary for feed intake reduction. However, increased concentrations of pro-inflammatory cytokines affect both orexigenic NPY/AgRP and anorexigenic POMC and derived α-MSH signalling in the hypothalamus. The classical view on the strict anorexic action of POMC neurons by producing α-MSH has recently been challenged as endocannabinoids shift the cleavage of POMC to the orexigenic peptide β-endorphin. Distinct endocannabinoids have anti-inflammatory properties. As both circulating endocannabinoids and hypothalamic β-endorphin concentrations increase in early lactation, the endocannabinoid system likely plays a key role in the modulation of feed intake and inflammation around calving. Targeting the pro-inflammatory and endocannabinoid signalling by optimising the availability and composition of dietary polyunsaturated fatty acids seems to be a promising approach to reduce the development of negative energy balance and metabolic disorders around calving.

## Software and data repository resources

None of the data were deposited in an official repository.
